# Response Surface Optimization of High-Durability Fly Ash–Slag Blended Concrete as an Eco-Friendly Repair Material

**DOI:** 10.3390/ma19061058

**Published:** 2026-03-10

**Authors:** Hua Wei, Anyi Chen, Chunhe Li, Jiaming Zhang, Hao Lu

**Affiliations:** 1 Materials & Structural Engineering Department, Nanjing Hydraulic Research Institutes, Nanjing 210029, China; hwei@nhri.cn (H.W.); chenanyi8959@163.com (A.C.); 2 Department of Civil and Environment Engineering, University of Miyazaki, Miyazaki 889-2192, Japan; lichunhe@cc.miyazaki-u.ac.jp; 3 College of Water Conservancy and Hydropower Engineering, Hohai University, Nanjing 210098, China

**Keywords:** eco-friendly materials, binary-blended concrete, response surface methodology, chloride transport resistance

## Abstract

To address the durability deficiencies and limited service life of concrete structures exposed to complex service environments such as chloride attack in marine and underground engineering, this study employs fly ash (FA) and ground granulated blast-furnace slag (GGBS), typical eco-friendly materials, as functional mineral admixtures to systematically investigate the effects of their combined incorporation on the mechanical properties, durability, drying shrinkage, and microstructural characteristics of concrete. The objective is to develop a concrete material that achieves high durability while maintaining structural safety and service performance, with the additional benefit of improved resource utilization efficiency. Single-factor tests were first conducted to determine the sensitivity ranges of FA and GGBS within 10–30% for slump, compressive strength, chloride migration coefficient (RCM), and drying shrinkage. Subsequently, response surface methodology (RSM) was employed to establish quadratic regression models using FA and GGBS as independent variables and compressive strength, RCM, and drying shrinkage as response indicators. The models exhibited high fitting accuracy, and their reliability was validated through analysis of variance (ANOVA), residual analysis, and predictive performance indices. Multi-objective optimization based on the desirability function identified the optimal mix proportion as FA = 14.8% and SL = 29.3%, yielding predicted values of 56.2 MPa for 28-day compressive strength, 6.03 × 10^−12^ m^2^/s for RCM, and 639 με for 90-day drying shrinkage. Microstructural analysis using SEM and MIP further revealed that the binary-blended system promotes the formation of a dense C–S–H/C–A–S–H gel network, refines pore-size distribution, and reduces pore connectivity, thereby improving long-term mechanical and durability performance. The findings provide quantitative guidance for designing high-durability, environmentally friendly concrete suitable for marine and underground engineering applications.

## 1. Introduction

Concrete is one of the most widely used construction materials in hydraulic and infrastructure engineering, particularly in harsh exposure conditions such as marine and underground environments. Nevertheless, when exposed to coastal environments, reinforced concrete is subjected to highly aggressive conditions characterized by elevated concentrations of chlorides and sulfates, coupled with carbonation, wetting–drying cycles, temperature fluctuations, and tidal actions. Similarly, tunnels and other underground hydraulic structures face severe durability challenges from groundwater ingress, internal water seepage, and chemical attacks. These combined environmental stresses accelerate steel corrosion, promote crack initiation and propagation, and ultimately lead to severe degradation of mechanical performance and service life reduction [1,2]. For massive or deeply embedded hydraulic structures, once deterioration such as cracking, spalling, or leakage occurs, repair becomes technically challenging, economically costly, and sometimes even infeasible without significantly interrupting structural operation. Therefore, improving the long-term durability of concrete under marine exposure has become a critical research priority in civil and hydraulic engineering.

To enhance the durability of concrete in aggressive environments, various material modification approaches have been proposed, including the incorporation of polymer latexes, resins, or other organic modifiers to improve pore structure and interfacial bonding performance [3]. However, such organic materials are often associated with relatively high costs, limited long-term durability, and poor construction adaptability. Moreover, the production and application processes of these materials may impose additional environmental burdens, restricting widespread use in large-scale projects and long-term service structures [4].

In contrast, regulating the hydration process and microstructural development of cementitious systems through the incorporation of mineral admixtures represents a more stable and engineering-feasible approach to durability enhancement. Among these, fly ash and slag, as typical industrial by-products, exhibit significant eco-friendly characteristics because their resource utilization can effectively replace part of cement, thereby reducing the high energy consumption and carbon emissions associated with cement production. In terms of microscopic mechanisms, FA contributes to pore refinement through its micro-filler effect and pozzolanic reaction, where amorphous silica reacts with calcium hydroxide to form additional C–S–H gel, thereby improving the density and homogeneity of the hardened matrix [5,6,7,8,9]. GGBS, on the other hand, possesses stronger latent hydraulic reactivity and can participate actively in early hydration, producing low Ca/Si ratio C–S–H and enhancing the compactness of the interfacial transition zone (ITZ) [10,11,12]. Numerous studies have confirmed that an appropriate dosage of FA and GGBS can effectively reduce permeability, inhibit chloride ingress, slow reinforcement corrosion, and improve long-term compressive strength [13,14,15]. Therefore, the introduction of a fly ash–slag blended system in durability-oriented mix design provides a feasible pathway for developing concrete materials that simultaneously achieve high structural performance and improved environmental compatibility.

While extensive research has been conducted on the individual effects of FA or GGBS on concrete performance, most of these studies remain confined to empirical observations or single-factor experiments. Such approaches fail to adequately reveal the potential nonlinear synergistic effects when these two materials are combined, and offer limited guidance for the coordinated optimization of multiple performance targets—particularly the simultaneous enhancement of mechanical strength, chloride penetration resistance, and volume stability—a critical requirement for concrete exposed to marine environments. Furthermore, systematic investigations into the coupled influence of FA-GGBS binary systems on the macroscopic properties of concrete under marine exposure conditions remain scarce [16,17,18,19,20,21,22]. To address these research gaps, this study employs a central composite design (CCD) within RSM framework, using FA and GGBS dosages as the primary variables to establish quadratic regression models. This approach enables not only the quantification of individual effects and their interactions, but also the identification of optimal mixture proportions through multi-objective optimization. Specifically, this study aims to: (1) systematically evaluate the coupled effects of FA and GGBS on compressive strength, chloride migration coefficient, and drying shrinkage; (2) establish predictive models capable of capturing the nonlinear behavior of the binary system; and (3) determine the optimal FA-GGBS combination that simultaneously enhances strength and durability while controlling shrinkage. By partially replacing cement with industrial by-products, this study provides both technical guidance and quantitative support for the development of high-durability concrete suitable for harsh marine and hydraulic environments, as well as for the resource utilization of solid wastes.

## 2. Materials and Methods

### 2.1. Materials

The cement used in this study was P·O 42.5 ordinary Portland cement produced by Anhui Conch Group (Wuhu, China). Its properties were tested in accordance with General Portland Cement [23]. The setting time, as well as the compressive and flexural strengths of cement mortar, were measured, and the physical and mechanical properties are presented in Table 1.

The fly ash used was Class I fly ash (Nanjing, China), and its physical properties are listed in Table 2.

The slag powder was Grade S95 GGBS (Nanjing, China), and its tested physical properties are shown in Table 3.

Table 4 presents the chemical compositions of the FA and GGBS used in this study. “Others” for FA includes minor oxides such as TiO_2_, K_2_O, Na_2_O, and P_2_O_5_.

The fine aggregate used in the experiment was Yangtze River medium sand (Nanjing, China), with a fineness modulus of 2.67, an apparent density of 2640 kg/m^3^, and a mud content of 1.0%, meeting the requirements of GB/T 14684-2022 Sand for Construction [26]. The coarse aggregates were continuously graded crushed stones (Nanjing, China), consisting of 20–40 mm gravel and 5–20 mm gravel. The apparent density, crushing value, and other properties of the coarse aggregates are provided in Table 5.

### 2.2. Test Program

#### 2.2.1. Single-Factor Tests

The single-factor tests were conducted using a unified baseline mix design with a fixed water-to-binder ratio (W/B) of 0.35, a sand ratio of 40%, and a superplasticizer dosage of 0.5% by mass of the binder. The replacement levels of FA and GGBS were varied independently from 0% to 40%, while all other constituents remained unchanged. The primary test indices included slump, compressive strength, flexural strength, chloride migration coefficient (RCM), and drying shrinkage. The single-factor results were used to determine the appropriate range for the binary-blended system. It was observed that the most significant performance variations occurred when FA and GGBS were within 10–30%, and no severe deterioration was detected; therefore, this interval was selected as the basis for factor levels in the response surface experiments.

#### 2.2.2. Response Surface Experiment

Based on the optimal dosage interval identified in the single-factor tests, a response surface methodology (RSM) experiment using the Central Composite Design was conducted. FA content (A) and GGBS content (B) were selected as the two independent variables. Their actual levels were set at 10%, 20%, and 30%, encoded as −1, 0, and +1, respectively. The CCD consisted of 13 experimental runs, including 4 axial points, 4 star points, and 5 center points.

#### 2.2.3. Sample Preparation and Mix Proportions

The concrete mixing procedure was as follows: cement, FA, GGBS, sand, and coarse aggregates were dry-mixed for 30 s; mixing water and superplasticizer were added and mixed for another 60 s; finally, the mixture was blended at high speed for 60 s to ensure homogeneity. After mixing, the fresh concrete was immediately cast into molds and compacted. Specimens were demolded after 24 h and cured under standard conditions at (20 ± 2) °C and relative humidity ≥95% until the designated testing ages.

Based on the CCD matrix, 13 concrete mixtures were prepared with FA and GGBS contents as variables. The detailed mix proportions for all tested samples, including the specific quantities of cement, FA, GGBS, sand, coarse aggregate, water, and superplasticizer, are presented in Table 6. All mixtures maintained a constant water-to-binder ratio of 0.35, sand ratio of 40%, and superplasticizer dosage of 0.5% by mass of binder.

### 2.3. Test Methods

#### 2.3.1. Workability Test

The slump and slump-flow tests were conducted in accordance with GB/T 50080-2016 [27]. Prior to testing, the slump cone and base plate were moistened. Fresh concrete was filled into the cone in three layers, each layer rodded 25 times. After lifting the cone vertically, the slump was measured immediately, and the base plate was gently tapped to obtain the slump-flow spread. The diameters in two perpendicular directions were recorded, and their average value was taken as the final result.

#### 2.3.2. Mechanical Properties Test

The compressive strength test was performed according to GB/T 50081-2019 [28] using 150 mm cube specimens. A YAW-3000 electro-hydraulic servo pressure testing machine (Xinheng Testing Equipment Co., Ltd., Hangzhou, China) with a maximum capacity of 3000 kN was used for testing. Three specimens were tested for each mix, and the average value was taken as the compressive strength. The loading rate was controlled at 18–30 MPa/min. The compressive strength was calculated using Equation (1):

(1) $$f_{cc}=\frac{P}{A}\times 1000$$

where f_cc_ is the compressive strength (MPa), P is the failure load (kN), and A is the loaded area of the specimen (mm^2^).

#### 2.3.3. Chloride Migration Coefficient Test

The non-steady-state chloride migration coefficient was determined using the RCM method [29] with an RCM-NTB chloride migration coefficient testing system (Niumat Testing Equipment Co., Ltd., Beijing, China). After specimen preparation, all samples were vacuum-saturated and sealed in silicone sleeves. The test setup consists of an anolyte (KOH solution), a catholyte (NaCl–KOH solution), and a DC power supply. A constant voltage of (30 ± 1) V was applied, and the initial current was recorded to determine the required test duration. After completion, the specimen was split and sprayed with 0.1 mol/L AgNO_3_ to visualize the chloride penetration depth. The average value of three specimens was taken as the final result; any individual value deviating more than 15% from the median was discarded.

#### 2.3.4. Drying Shrinkage Test

Following SL/T 352-2020 [29], prism specimens with dimensions 100 mm × 100 mm × 515 mm were prepared. Stainless-steel gauge studs were embedded at both ends of the mould. After casting, specimens were cured for 1 day, demoulded, and immediately transferred to the drying-shrinkage chamber (temperature 20 ± 2 °C, relative humidity 60 ± 5%). The initial length L_0_ was recorded, and subsequent length measurements were taken at 3, 7, 14, 28, 60, and 90 days. Three specimens were tested for each mix, and the average value was calculated. Shrinkage strain was computed using Equation (2):

(2) $$\varepsilon_{t}=\frac{\Delta L_{t}}{L}$$

where *ε*_*t*_ is the shrinkage strain at age *t*, Δ*L*_*t*_ is the length change at age *t* (mm), *L* is the effective gauge length (mm), *L*_*t*_ is the measured length at age *t*, *L*_0_ is the initial reference length.

#### 2.3.5. Microstructural Analysis Methods

SEM was used to observe the morphology of hydration products in the reference concrete and the optimum FA–GGBS blend at 28 days. After compressive testing, fragments approximately 10 mm × 10 mm × 10 mm were extracted from the interior of the specimens. The samples were immersed in anhydrous ethanol at 28 days to stop hydration, dried, sputter-coated with gold, and subsequently examined under a QUANTA 250 FEG scanning electron microscope (FEI Company, Hillsboro, OR, USA).

Mercury intrusion porosimetry (MIP) was employed to analyze the pore structure of the reference and optimum blended concretes. After the 28-day compressive test, fragments approximately 5 mm × 5 mm × 5 mm were taken from the paste matrix and ITZ regions, avoiding coarse aggregates. Hydration was terminated by immersion in anhydrous ethanol for 24 h, followed by vacuum drying at 50 °C to constant mass. The dried samples were tested in an AutoPore IV 9500 mercury intrusion porosimeter (Micromeritics Instrument Corporation, Norcross, GA, USA) to obtain pore size distribution, most probable pore size, threshold pore diameter, and total porosity, providing insights into the pore-refinement effects of the FA–GGBS blended system.

## 3. Results and Discussion

### 3.1. Single-Factor Analysis

#### 3.1.1. Fly Ash Content

**Figure 1:**
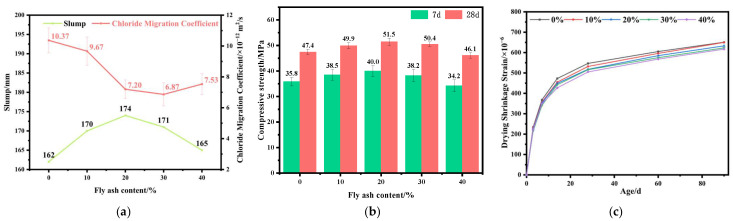
Influence of fly ash content on the properties of concrete: (**a**) slump and chloride diffusion coefficient; (**b**) compressive strength; (**c**) drying shrinkage.

The incorporation of FA leads to a noticeable modification in the flow characteristics of the fresh mixture. As shown in Figure 1a, the slump increases rapidly at low replacement levels (0–20%), rising from 162 mm in the reference mixture to 174 mm. This improvement is primarily attributed to the smooth surface and near-spherical morphology of FA particles, which provide a “ball-bearing” effect that reduces the internal friction of the paste [30]. When the replacement level exceeds 20%, the slump begins to decline, decreasing to 165 mm at 40%, though still slightly higher than the reference group. This reversal is mainly due to excessive FA absorbing mixing water and retarding hydration, which increases paste viscosity and reduces flowability. Overall, FA enhances workability at low to moderate dosages, but this beneficial effect diminishes and eventually reverses when the content exceeds approximately 30%.

The chloride diffusion coefficient also exhibits a clear dependence on FA content. The reference mixture shows an average value of 10.4 × 10^−12^ m^2^/s; with 10% FA, the coefficient decreases to approximately 9.67 × 10^−12^ m^2^/s, and reaches its minimum value at 30% replacement (6.87 × 10^−12^ m^2^/s), indicating that chloride transport pathways within the interfacial transition zone are effectively blocked at this dosage [14]. When the replacement exceeds 30%, the diffusion coefficient increases again, suggesting that excessive FA reduces the amount of effective cementitious hydrates and increases pore connectivity.

As shown in Figure 1b, the compressive strength exhibits a staged improvement with the incorporation of FA. At 7 days, the reference strength is 35.8 MPa; mixtures with 10–20% FA show increased strength, with the maximum improvement reaching 11.7%, while the 30–40% groups show slightly lower values compared with the peak. At 28 days, as the pozzolanic reaction of FA continues, the strength increases overall, with the peak occurring at 20% replacement, reaching 51.5 MPa—an 8.6% increase over the reference. When the dosage reaches 40%, excessive dilution of cement clinker slows hydration [31,32], reducing the strength to approximately 46.1 MPa. It is noteworthy that although high FA mixtures show lower early-age strength, their later-age strength development is faster, indicating a compensatory effect from the prolonged pozzolanic reaction [17].

The evolution of drying shrinkage strain shows a similar trend across all FA dosages, with rapid growth during the early age (0–14 days) and gradual stabilization thereafter. As shown in Figure 1c, the 90-day shrinkage of the reference mixture is 651 × 10^−6^. Compared with this value, the mixtures containing 10–40% FA showed reductions of 8.5%, 11.1%, 17.3%, and 24.3%, respectively. FA effectively mitigated drying shrinkage by refining the pore structure through micro-filling and pozzolanic reactions, reducing pore connectivity, and slowing internal moisture loss.

#### 3.1.2. GGBS Content

**Figure 2:**
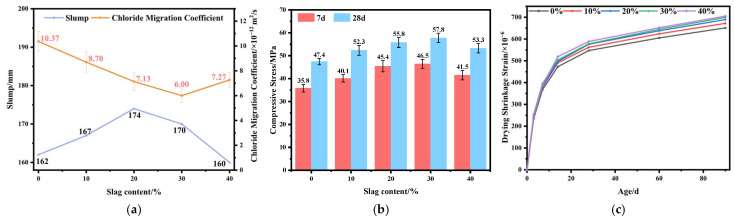
Influence of slag content on the properties of concrete: (**a**) slump and chloride diffusion coefficient; (**b**) compressive strength; (**c**) drying shrinkage.

The slump of concrete exhibits an overall trend of first increasing and then decreasing with the incorporation of GGBS. As shown in Figure 2, the slump of the reference mixture is 162 mm; when the GGBS replacement level reaches 20%, the slump attains its peak value of 174 mm. This improvement is attributed to the smooth particle surface and spherical morphology of GGBS, which enhance the flowability of the mixture through a ball-bearing effect. However, at a replacement level of 40%, the slump decreases to 160 mm, indicating that excessive GGBS increases the water demand of the system and the entrapped air content, thereby reducing workability. Overall, a moderate dosage of GGBS (approximately 10–30%) is beneficial to improving the workability of fresh concrete, while excessive incorporation leads to slump loss.

The chloride migration coefficient *D*_RCM_ decreases initially and then increases with increasing GGBS content. The reference mixture shows an average value of 10.4 × 10^−12^ m^2^/s. When the replacement level reaches 30%, the RCM value drops to its minimum of 6.0 × 10^−12^ m^2^/s, representing a 42.1% reduction compared with the baseline. This indicates that the pozzolanic reaction and micro-filling effect of GGBS significantly enhance the matrix densification [10]. Further increasing the GGBS content to 40% results in an increase in the RCM value to approximately 7.3 × 10^−12^ m^2^/s, suggesting that excessive GGBS reduces the volume of effective hydrates and increases pore connectivity. Although some variability exists among parallel specimens (CV ≈ 8–15%) and slight overlap occurs between certain data points, the overall trend remains consistent, with the optimal range around 30%.

Both the 7-day and 28-day compressive strengths follow a typical “increase–peak–decrease” pattern as the GGBS content increases. The reference mixture exhibits a 28-day strength of 47.4 MPa; the strength reaches a maximum of 57.8 MPa at a 30% replacement level, corresponding to a 21.9% improvement, and decreases to 53.3 MPa at 40%. The trend is consistent with that observed at early ages, indicating that an appropriate amount of GGBS improves pore structure and enhances later-age hydration, whereas excessive GGBS dilutes cement clinker and slows strength development.

Drying shrinkage strain increases continuously with age for all mixtures, showing rapid growth within 0–14 days and stabilizing after 28 days. With prolonged curing, mixtures containing 10–40% GGBS show higher shrinkage than the reference, indicating that the incorporation of GGBS intensifies volume contraction following hydration heat release. The S = 40% mixture exhibits the highest 90-day shrinkage, reaching 705 × 10^−6^. From a microstructural perspective, the increased drying shrinkage in GGBS-blended concrete can be attributed to the refinement of the pore structure. The high reactivity of GGBS promotes the formation of additional C–S–H gel, which fills larger capillaries and transforms them into a greater number of finer pores. While this pore refinement is beneficial for mechanical properties and impermeability, it simultaneously intensifies self-desiccation. As internal relative humidity drops, finer pores generate higher capillary tensile stresses in the pore water, leading to greater bulk shrinkage of the concrete matrix [16]. This mechanism contrasts with that of fly ash, which, due to its slower pozzolanic reaction, contributes less to early-age pore refinement and self-desiccation, thus exhibiting a mitigating effect on drying shrinkage as observed in Figure 1c.

### 3.2. Response Surface Design and Analysis

#### 3.2.1. Model Establishment

After identifying the fundamental effects of FA and GGBS on the mechanical and durability properties of concrete, the response surface methodology (RSM) was further employed to perform multi-factor optimization of the binary-blended system. The FA replacement level (A) and GGBS replacement level (B) were selected as the primary independent variables, while the response variables included the 28-day compressive strength (Y_1_), the 28-day chloride migration coefficient (Y_2_), and the 90-day drying shrinkage strain (Y_3_). To ensure consistent hydration conditions across all mixtures, the water-to-binder ratio was fixed at 0.35.

Based on the preceding single-factor sensitivity analysis, variations in FA and GGBS between 10% and 30% produced the most significant influence on concrete performance. Accordingly, the coded factor levels of −1, 0, and +1 were adopted to represent actual replacement levels of 10%, 20%, and 30%, respectively. A second-order polynomial model involving linear terms, interaction terms, and quadratic terms was employed to construct the response surfaces. A total of 13 experimental runs were generated, including five replicated center-point tests to estimate pure error and evaluate model lack of fit. The experimental design and corresponding test results are summarized in Table 7.

Based on the experimental data, the quadratic polynomial regression models for the three performance indicators were obtained as follows:

(3) $$Y_{1}=53.13-2.00A+3.00B-0.875AB-2.521A^{2}+0.579B^{2}$$

(4) $$Y_{2}=6.645+0.25A-0.933B+0.20AB+0.743A^{2}+0.093B^{2}$$

(5) $$Y_{3}=662.86+17.50A-24.17B+6.25AB+20.983A^{2}-2.017B^{2}$$

All three regression models contain significant quadratic terms, indicating that the effects of FA and GGBS on concrete performance exhibit clear nonlinear behavior. The curvature of the response surfaces reflects the combined influence of linear, interaction, and quadratic components, confirming that the binary-blended system does not follow a simple proportional response with respect to admixture content.

#### 3.2.2. ANOVA and Significance Analysis

To evaluate the statistical significance and predictive capability of the developed quadratic regression models, an analysis of variance (ANOVA) was performed for each response variable. ANOVA examines the ratio between the mean square of a model term and that of the residual error (F-value) to determine the significance of the regression relationship. The corresponding *p*-values quantify the confidence level, with smaller values indicating stronger statistical significance.

Table 8, Table 9 and Table 10 summarize the ANOVA results for the quadratic regression models of compressive strength, chloride migration coefficient, and drying shrinkage, respectively.

For the Y_1_ (compressive strength) model, both factors A (FA content) and B (GGBS content) exhibit significant effects, with *p*-values of 0.00088 and 0.000072. This indicates that both admixtures play a meaningful role in strength development, with GGBS contributing more prominently. The quadratic term A^2^ is also significant (*p* = 0.00213), demonstrating a pronounced nonlinear effect of FA on strength. In contrast, the B^2^ term is not significant, suggesting that GGBS does not introduce a comparable nonlinear influence.

For the Y_2_ (chloride migration coefficient) model, the GGBS content (B) exhibits a highly significant effect (*p* = 0.000053), confirming its critical role in reducing ionic transport. The FA content (A) shows a marginally significant influence (*p* = 0.0526). The quadratic term A^2^ is significant (*p* = 0.00221), indicating that changes in FA dosage introduce nonlinear variations in chloride transport behavior. Neither the interaction term AB nor the B^2^ term is significant, suggesting that no statistically significant synergistic effect was detected between FA and GGBS for this durability indicator within the experimental range studied.

In the Y_3_ (drying shrinkage) model, both A (*p* = 0.00021) and B (*p* = 0.000026) show extremely significant effects, with GGBS exerting a stronger influence. The quadratic term A^2^ is significant (*p* = 0.000734), implying that FA induces a nonlinear variation in drying shrinkage. Meanwhile, B^2^ remains insignificant, indicating a weaker nonlinear contribution from GGBS.

To further assess model adequacy and predictive performance, the coefficients of determination (R^2^), adjusted coefficients of determination (Adjusted R^2^), and predicted coefficients of determination (Predicted R^2^) were calculated based on the residual sum of squares, pure error from replicated center points, and PRESS statistics. The results are summarized in Table 11.

The three models exhibit R^2^ values of 0.9475, 0.9412, and 0.9631; Adjusted R^2^ values of 0.9100, 0.8992, and 0.9367; and Predicted R^2^ values of 0.6561, 0.6195, and 0.7545, respectively. The differences between Adjusted R^2^ and Predicted R^2^ for all responses are below 0.20, indicating that the models do not suffer from overfitting and possess stable predictive capability.

Overall, the ANOVA results confirm that all three regression models are statistically significant and capable of accurately characterizing the effects of FA–GGBS binary blending on the mechanical and durability performance of concrete.

#### 3.2.3. Model Reliability and Residual Analysis

To further verify the robustness of the regression models, the pure error was first estimated from the replicated center-point tests, and the lack-of-fit (LOF) term was subsequently evaluated by comparing the pure error with the residual sum of squares. As shown in Table 9, the LOF F-values for Y_1_, Y_2_, and Y_3_ are 4.30, 11.07, and 15.13, respectively. None of these values reach statistical significance, indicating that the model errors primarily originate from experimental noise rather than structural deficiencies of the regression equation. This confirms that the quadratic polynomial form is appropriate for representing the FA–GGBS binary blending system.

**Figure 3:**
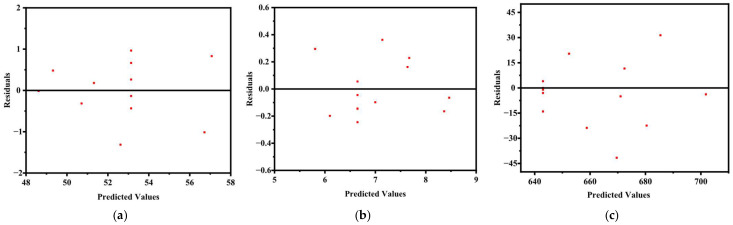
Scatter plot of residuals versus fitted values: (**a**) Y_1_; (**b**) Y_2_; (**c**) Y_3_.

In addition, the residual–fitted value scatter plots for the three responses (Figure 3) further support the adequacy of the models. The residuals are randomly distributed around zero without displaying a funnel-shaped pattern, suggesting that the assumption of homoscedasticity is satisfied. The absence of systematic deviation indicates that no key variable or interaction effect has been omitted from the model.

The predictive capability of the regression models was also assessed based on the Predicted R^2^ values presented in Table 3, Table 4, Table 5 and Table 6, which are 0.6561, 0.6195, and 0.7545 for Y_1_, Y_2_, and Y_3_, respectively. These values demonstrate acceptable predictive performance and confirm that the models possess adequate external validity. Taken together, the lack-of-fit evaluation, residual diagnostics, and predictive statistics indicate that all three regression models are statistically reliable and suitable for subsequent response surface analysis and optimization.

#### 3.2.4. Response Surface and Interaction Effects Analysis

**Figure 4:**
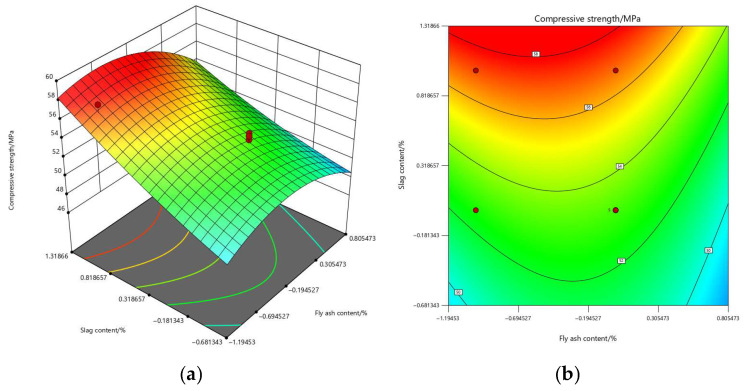
Response surface and contour plots of compressive strength: (**a**) response surface; (**b**) contour map.

**Figure 5:**
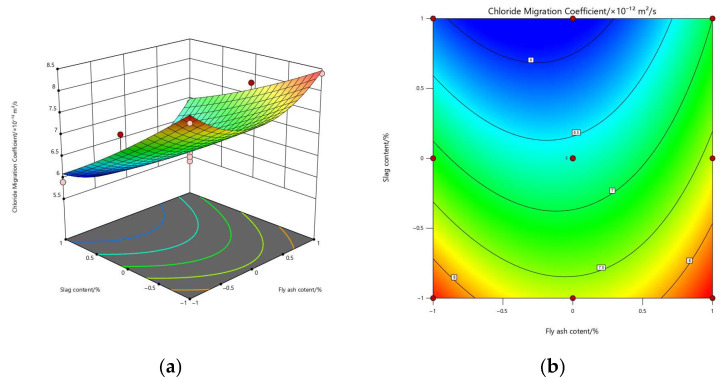
Response surface and contour plots of chloride migration coefficient: (**a**) response surface; (**b**) contour map.

**Figure 6:**
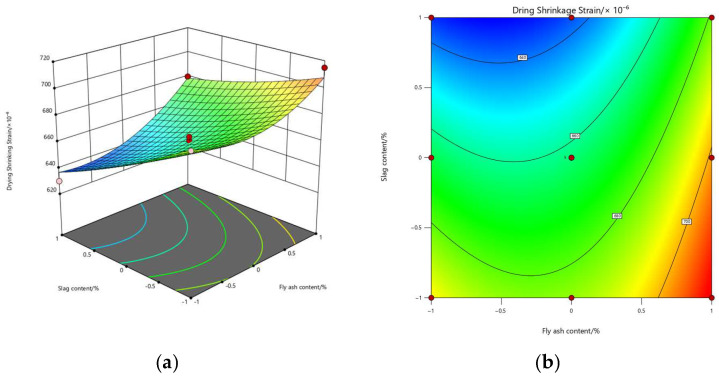
Response surface and contour plots of drying shrinkage: (**a**) response surface; (**b**) contour map.

Based on the fitted quadratic polynomial regression models, three-dimensional response surface plots and contour maps were constructed to illustrate the effects of FA (A) and GGBS (B) on the three performance indicators, as shown in Figure 4, Figure 5 and Figure 6. These plots provide an intuitive representation of the main effects, quadratic effects, and potential interaction effects within the binary-blended system.

From the overall morphology of the response surfaces, all three performance indicators exhibit distinct curvature, which is consistent with the ANOVA results. This confirms that the nonlinear behavior of the system is primarily governed by the quadratic term of FA (A^2^), whereas the nonlinear contribution of GGBS (B^2^) is comparatively weaker. The transition from planar to curved surfaces further verifies that quadratic components play a substantial role in governing the performance variations.

For compressive strength (Y_1_), the response surface displays a “mountain-peak” pattern, with the optimum region observed at low–to–moderate FA contents and relatively high GGBS contents (approximately FA = 15–20%, GGBS = 25–30%). Pronounced slopes appear along both factor directions, with a steeper gradient in the GGBS direction, indicating that GGBS contributes more significantly to strength enhancement. This observation aligns with the ANOVA findings, where the *p*-value corresponding to B was considerably smaller than that of A. Moreover, the downward curvature in the FA direction reflects the significance of the A^2^ term (*p* = 0.00213), indicating that excessive FA leads to a noticeable reduction in strength.

The response surface of the chloride migration coefficient (Y_2_) exhibits a “saddle-shaped descending” pattern, with the optimal performance region occurring at medium-to-low FA contents and high GGBS contents. With increasing GGBS dosage, Y_2_ decreases markedly, whereas the influence of FA is relatively weaker and only slightly nonlinear. This behavior is in full agreement with the ANOVA results: B demonstrates extremely high significance (*p* = 0.000053), while A is only marginally significant (*p* = 0.0526). The absence of pronounced twisting or skewing on the surface indicates that the AB interaction does not exert a meaningful synergistic effect on ion transport, consistent with the non-significant AB term (*p* = 0.172).

For drying shrinkage (Y_3_), the response surface shows a smooth but clearly curved profile. Shrinkage increases significantly at high FA contents and decreases noticeably at high GGBS contents, producing a “tilted valley” shape. The curvature along the FA direction is the most prominent, corresponding to the high significance of the A^2^ term (*p* = 0.000734), indicating strong nonlinear influence of FA on volume stability. The steeper slope in the GGBS direction reflects the dominant role of GGBS in reducing shrinkage (*p* = 0.000026). Again, the absence of surface distortion corroborates the non-significance of the AB interaction term.

Overall, the response surfaces collectively reveal that the interaction between FA and GGBS is weak across all three performance indicators, as evidenced by the lack of complex cross-curvature or inclined valley structures. In summary, FA primarily introduces nonlinear curvature effects, while GGBS exhibits a more pronounced linear main effect across all responses. The contour maps further identify that the optimal performance region consistently appears in the “low-FA, high-GGBS” quadrant, which is fully consistent with the multi-objective optimization results where the optimal solution was obtained at approximately FA ≈ 15% and GGBS ≈ 29%.

### 3.3. Multi-Objective Optimization Analysis

Based on the quadratic regression models developed in the previous section, a multi-objective response surface optimization was conducted to determine the optimal combination of FA and GGBS in the binary-blended concrete system. The optimization objectives were to simultaneously increase compressive strength, reduce the chloride migration coefficient, and minimize drying shrinkage strain. To ensure practical applicability in engineering practice, the ranges of FA and GGBS were constrained to 10–30%.

A weighted desirability function approach was employed to integrate the three performance indicators into a single composite desirability index *D*. Each response was normalized according to its target direction—“larger-the-better” for compressive strength and “smaller-the-better” for chloride migration coefficient and drying shrinkage. Equal weights were assigned to all three responses (weight = 1) to balance mechanical performance and durability performance without bias. The optimization process was then performed by maximizing the overall desirability function within the FA–GGBS design domain.

The optimization results indicate that the region corresponding to slightly lower FA content and relatively higher GGBS content provides the most favorable combined performance. The optimal mixture predicted by maximizing the composite desirability is: FA = 14.8%; GGBS = 29.3%. The predicted performance indicators at this optimum point are: 28-day compressive strength: approximately 56.2 MPa; Chloride migration coefficient: approximately 6.03 × 10^−12^ m^2^/s; 90-day drying shrinkage strain: approximately 639 με.

The optimized mixture (FA = 14.8%, GGBS = 29.3%) represents a state of microstructural equilibrium, in which FA and GGBS achieve synergistic enhancement of macroscopic performance. The high GGBS content drives the formation of a dense C-S-H gel network through its latent hydraulic reactivity. On one hand, the C-S-H gel directly constitutes the strength skeleton of the concrete, significantly improving compressive strength. On the other hand, the gel fills capillary pores and refines the pore structure, effectively blocking the transport channels for chloride ion penetration, thereby substantially reducing the chloride migration coefficient. However, the pore refinement induced by GGBS also increases drying shrinkage. This is because a finer pore structure generates higher capillary tensile stresses in the pore water as internal relative humidity decreases, thereby intensifying self-desiccation shrinkage. In this equilibrium, the incorporation of 14.8% FA effectively mitigates drying shrinkage, which can be primarily attributed to its slower pozzolanic reaction that reduces early-age autogenous shrinkage, as well as its micro-filler effect that optimizes the pore structure without significantly increasing capillary tension. Consequently, in this binary system, the shrinkage-inducing effect of the high GGBS content is effectively counteracted by the shrinkage-mitigating effect of FA. The resulting drying shrinkage strain is controlled at 639 με, which is even lower than that of the plain cement reference (651 με), while both compressive strength (56.2 MPa) and chloride penetration resistance (6.03 × 10^−12^ m^2^/s) are significantly enhanced.

### 3.4. Microstructural Analysis

#### 3.4.1. SEM Analysis

**Figure 7:**
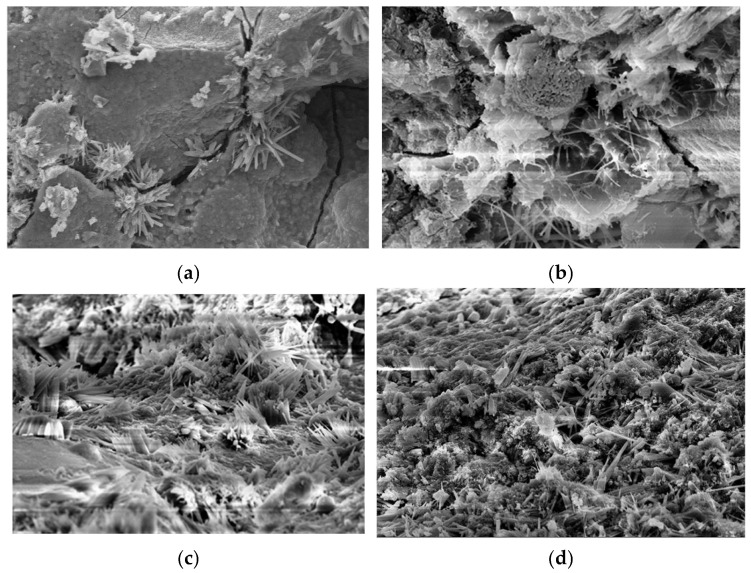
SEM images: (**a**,**b**) reference group; (**c**,**d**) optimal FA–GGBS binary-blended mixture. Magnification: 4.24 KX for (**a**), 21.23 KX for (**b**), 10.0 KX for (**c**,**d**).

As shown in Figure 7a,b, the reference specimen exhibits pronounced internal cracks. These cracks penetrate the paste matrix, and their edges present a flaked and brittle morphology, indicating weak internal connectivity and a tendency for microcracks to propagate along vulnerable interfaces. Numerous plate-like crystals, typically identified as Ca(OH)_2_, and dispersed needle-like hydration products (such as C–S–H or AFt) can be observed on both sides of the cracks. However, these hydration products do not form a continuous or compact gel network; instead, they are loosely arranged with large voids. The rough surface and the presence of abundant unhydrated particles further indicate insufficient hydration and high pore connectivity within the matrix.

In contrast, Figure 7c,d show a large quantity of fine fibrous and flaky gels arranged in a continuous, interwoven manner, forming a uniform and dense gel network. These gel phases are typically a mixture of C-S-H and C-A-S-H. Compared with the reference group, the number of needle-clustered and dendritic gels in the optimal FA–GGBS blended system is significantly higher, with smaller dimensions and tighter packing, suggesting that the secondary hydration induced by FA and GGBS is well-developed. The inter-gel pores are extremely limited, and no penetrating microcracks or large voids are observed, demonstrating a substantial reduction in connected pores and a refined pore structure [33]. This dense and continuous gel network effectively fills capillary pores and refines the pore structure, thereby blocking chloride penetration pathways and significantly reducing the chloride migration coefficient.

#### 3.4.2. MIP Analysis

**Figure 8:**
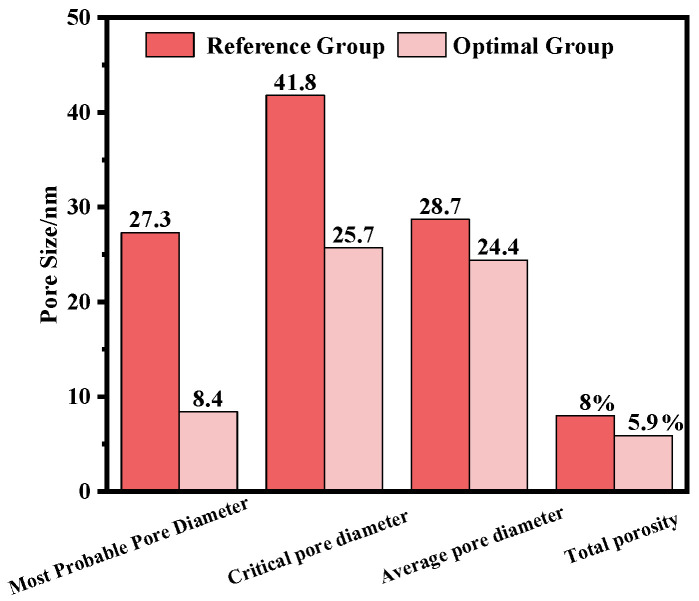
Comparison of pore-structure parameters between the reference mix and the optimal FA–GGBS blend.

The pore structure of concrete can be characterized using parameters such as the most probable pore diameter, critical pore diameter, average pore diameter, and total porosity. These parameters reflect the dominant pore size, the connectivity of transport pathways, and the overall pore refinement. The MIP results are presented in Figure 8.

For the reference concrete, the most probable pore diameter is 27.3 nm, the critical pore diameter is 41.8 nm, the average pore diameter is 28.7 nm, and the total porosity reaches 8.0%. In contrast, the optimal FA–GGBS mixture shows substantial reductions in all pore structure indicators, with the most probable pore diameter decreasing to 8.4 nm, the critical pore diameter to 25.7 nm, the average pore diameter to 24.4 nm, and the total porosity to 5.9%.

These reductions indicate that the pore structure of the FA–GGBS blended concrete becomes significantly refined and less connected, consistent with the dense gel network observed in the SEM images. This microstructural improvement provides a mechanistic explanation for the enhanced resistance to chloride ingress observed in the blended system [34].

## 4. Conclusions

(1) Single-factor test results indicate that FA exerts a more pronounced regulatory effect on workability and drying shrinkage, whereas GGBS is more conducive to enhancing compressive strength and chloride migration resistance. Both materials exhibit a nonlinear response characterized by “enhancement at moderate dosages and deterioration at excessive dosages,” suggesting that their synergistic effect exists within an optimal dosage range. This provides critical guidance on threshold dosages for the resource utilization of industrial solid wastes.

(2) The quadratic regression models developed using response surface methodology demonstrate excellent goodness-of-fit (R^2^ > 0.94) and statistical significance, enabling the quantitative characterization of the interactive effects of FA and GGBS on the strength, durability, and shrinkage properties of concrete. The high agreement between model predictions and experimental results verifies the reliability of this method for the multi-objective mix design of eco-friendly concrete materials.

(3) Multi-objective optimization reveals that the FA–GGBS binary system achieves synergistic enhancement of macroscopic properties within the “low FA–high GGBS” range (FA = 14.8%, GGBS = 29.3%): compressive strength reaches 56.2 MPa, chloride migration coefficient decreases to 6.03 × 10^−12^ m^2^/s, and drying shrinkage is controlled at 639 με. This optimized formulation demonstrates that partial replacement of cement with industrial solid wastes can still ensure the high performance and long-term serviceability required for structural materials.

(4) Microstructural mechanism analysis reveals that the performance improvement stems from the synergistic densification effect within the composite cementitious system. The optimized mixture forms a continuous and dense C–S–H/C–A–S–H gel network with an intact interfacial transition zone. The most probable pore diameter is refined from 27.3 nm to 8–10 nm, and the total porosity is reduced by approximately 25%. These findings elucidate, from a materials science perspective, the synergistic strengthening mechanism of FA and GGBS as eco-friendly cementitious materials, providing a theoretical foundation for their large-scale application in green high-performance concrete.

While this study provides a systematic optimization of the FA-GGBS binary system and elucidates its microstructural mechanisms, several aspects warrant further investigation. Firstly, future studies could extend the replacement ranges beyond 10–30% to investigate the behavior of FA-GGBS binary systems under extreme dosage conditions, particularly for applications requiring specialized performance characteristics. Secondly, long-term performance evaluation extending beyond 90 days to 1 year or more is recommended to validate the evolution of mechanical properties, chloride resistance, and shrinkage under sustained conditions. Thirdly, field validation studies are essential to assess the material’s performance under real-world exposure scenarios, such as tidal action in marine environments or groundwater pressure in tunnels, where coupled mechanical and environmental loads are present. Finally, the environmental and economic benefits of large-scale application of this optimized eco-friendly concrete could be quantified through life-cycle assessment (LCA) to further support its practical implementation.

## Figures and Tables

**Table 1:** Physical and Mechanical Properties of Cement.

Raw Materials	Water Requirement for Standard Consistency/%	Compressive Strength/MPa		Flexural Strength/MPa		Setting Time/min		Soundness
		3 d	28 d	3 d	28 d	Initial	Final	
P O 42.5	28.0	23.5	44.2	5.4	8.4	205	255	Pass
GB175-2023 [23]	——	≥17.0	≥42.5	≥4.0	≥6.5	≥45	≤600	Pass

**Table 2:** Physical Properties of Fly Ash.

Physical Properties	Density/g/cm^3^	Fineness/%	Water Requirement Ratio/%	Loss on Ignition/%	Activity Index/%
Fly Ash	2.51	3.6	95	1.84	78
Grade I Fly Ash (GB/T 1596-2017) [24]	≤2.6	≤12.0	≤95	≤5.0	≥70

**Table 3:** Physical Properties of GGBS.

Physical Properties	Moisture Content/%	Density/g/cm^3^	Specific Surface Area/m^2^/kg	Flow Ratio/%	Activity Index/%	
					7 d	28 d
Slag Powder	0.07	2.86	436	100	80	109
S95(GB/T18046-2017) [25]	≤1.0	≥2.8	≥400	≥95	≥75	≥95

**Table 4:** Chemical composition of FA and GGBS (wt%).

Material	SiO_2_	Al_2_O_3_	Fe_2_O_3_	CaO	MgO	SO_3_	LOI	Others	Total
FA	53.8	29.2	6.5	4.0	1.3	0.9	1.8	2.5	100.0
GGBS	34.8	15.2	1.2	39.5	8.0	1.2	0.1	-	100.0

**Table 5:** Main Performance Indicators of Coarse Aggregates.

Coarse Aggregate	Apparent Density/g/cm^3^	Mud Content/%	Mud Lumps Content/%	Crushing Value/%
Medium Gravel	2.70	0.04	0	12.5
Small Gravel	2.68	0.15	0	12.5

**Table 6:** Mix proportions of FA-GGBS binary-blended concrete (kg/m^3^).

Run	FA/%	GGBS/%	Cement	FA	GGBS	Sand	Coarse Aggregate	Water	SP
1	30	20	187.5	112.5	75	758	1136	131	1.88
2	10	30	225	37.5	112.5	758	1136	131	1.88
3	20	20	225	75	75	758	1136	131	1.88
4	20	30	187.5	75	112.5	758	1136	131	1.88
5	30	30	150	112.5	112.5	758	1136	131	1.88
6	10	10	300	37.5	37.5	758	1136	131	1.88
7	20	20	225	75	75	758	1136	131	1.88
8	10	20	262.5	37.5	75	758	1136	131	1.88
9	20	10	262.5	75	37.5	758	1136	131	1.88
10	30	10	225	112.5	37.5	758	1136	131	1.88
11	20	20	225	75	75	758	1136	131	1.88
12	20	20	225	75	75	758	1136	131	1.88
13	20	20	225	75	75	758	1136	131	1.88

**Table 7:** Experimental design and test results.

Run	FA/%	GGBS/%	A	B	Y_1_ (MPa)	Y_2_ (10^−12^ m^2^/s)	Y_3_ (10^−6^)
1	30	20	1	0	48.6	7.8	705
2	10	30	−1	1	57.9	5.9	630
3	20	20	0	0	53.4	6.5	660
4	20	30	0	1	55.7	6.1	642
5	30	30	1	1	51.5	6.9	680
6	10	10	−1	−1	49.8	8.2	690
7	20	20	0	0	54.1	6.6	659
8	10	20	−1	0	51.3	7.5	675
9	20	10	0	−1	50.4	7.9	692
10	30	10	1	−1	46.9	8.4	715
11	20	20	0	0	53	6.4	661
12	20	20	0	0	52.7	6.7	664
13	20	20	0	0	53.8	6.5	658

**Table 8:** ANOVA Results of the Y_1_ Regression Model.

Source	DF	SS	MS	F-Value	*p*-Value
A	1	24.00	24.00	30.60	0.00088
B	1	54.00	54.00	68.84	0.000072
AB	1	3.06	3.06	3.90	0.0887
A2	1	17.55	17.55	22.37	0.00213
B2	1	0.93	0.93	1.18	0.313
Residual	7	5.49	0.78	—	—

**Table 9:** ANOVA Results of the Y_2_ Regression Model.

Source	DF	SS	MS	F-Value	*p*-Value
A	1	0.375	0.375	5.43	0.0526
B	1	5.227	5.227	75.64	0.000053
AB	1	0.160	0.160	2.32	0.172
A2	1	1.525	1.525	22.07	0.00221
B2	1	0.024	0.024	0.35	0.575
Residual	7	0.484	0.069	—	—

**Table 10:** ANOVA Results of the Y_3_ Regression Model.

Source	DF	SS	MS	F-Value	*p*-Value
A	1	1837.50	1837.50	49.14	0.00021
B	1	3504.17	3504.17	93.70	0.000026
AB	1	156.25	156.25	4.18	0.0802
A2	1	1216.00	1216.00	32.52	0.000734
B2	1	11.24	11.24	0.30	0.600
Residual	7	261.77	37.40	—	—

**Table 11:** Statistical Summary of the Response Models.

Indicator	Y_1_	Y_2_	Y_3_
Pure Error SS	1.30	0.0520	21.20
Pure Error DF	4	4	4
LOF SS	4.19	0.4317	240.57
LOF DF	3	3	3
MS (PE)	0.325	0.0130	5.30
MS (LOF)	1.397	0.1439	80.19
F (LOF)	4.30	11.07	15.13
R^2^	0.9475	0.9412	0.9631
Adjusted R^2^	0.9100	0.8992	0.9367
Predicted R^2^	0.6561	0.6195	0.7545

## Data Availability

The original contributions presented in this study are included in the article. Further inquiries can be directed to the corresponding authors.

## References

[1] Elahi M.M.A., Shearer C.R., Naser Rashid Reza A., Saha A.K., Khan M.N.N., Hossain M.M., Sarker P.K. (2021). Improving the Sulfate Attack Resistance of Concrete by Using Supplementary Cementitious Materials (SCMs): A Review. Constr. Build. Mater..

[2] Zhang J., Song W., Wang H., Chen Z., Liu Q., Qi F. (2026). Experimental investigation and numerical study on chloride diffusion coefficient of long-term fly ash concrete. Case Stud. Constr. Mater..

[3] Wang R., Tian N., Li Y. (2026). Performance evolution and life prediction of different polymeric coatings for concrete under coupled action of sulfate attack and dry-wet cycles. J. Build. Eng..

[4] Wang K., Dong K., Guo J., Du H. (2024). Absorption and Release mechanism of superabsorbent polymers and its impact on shrinkage and durability of internally cured concrete—A review. Case Stud. Constr. Mater..

[5] Shi X., Xie N., Fortune K., Gong J. (2012). Durability of Steel Reinforced Concrete in Chloride Environments: An Overview. Constr. Build. Mater..

[6] Zhang J., Zhou X., Zheng J. (2020). Experimental Investigation and Analytical Modeling of Chloride diffusion coefficient of Fly Ash Concrete. Materials.

[7] Fernando P.T., João C.G., Said J. (2010). Durability and Environmental Performance of Alkali-Activated Tungsten Mine Waste Mud Mortars. J. Mater. Civ. Eng..

[8] Liu Z., Takasu K., Koyamada H., Suyama H. (2022). A Study on Engineering Properties and Environmental Impact of Sustainable Concrete with Fly Ash or GGBS. Constr. Build. Mater..

[9] Zhao Y., Wu Y., Hu D., Cai Y., Liu Y., Chen H. (2024). Study of Microstructure and Mechanical Properties and Residual Stresses of 24CrNiMo Steel Prepared by Selective Laser Melting and Laser Melting Deposition. J. Mater. Res. Technol..

[10] Oh S., Kim J., Song C., Choi S. (2024). Effects of Fineness and Substitution Rate of GGBFS on Material Characteristics of GGBFS-Blended Cement Mortars: Hydration, Non-Evaporable Water, Pore Structure, Mechanical Properties, Self-Desiccation, and Autogenous Shrinkage. J. Build. Eng..

[11] Ravina D., Mehta P.K. (1988). Compressive Strength of Low Cement/High Fly Ash Concrete. Cem. Concr. Res..

[12] Oh S., Oh G., Hong G., Choi Y.-C., Choi S. (2025). Thermomechanical Properties of High-Volume Fly Ash Concrete for Application in Mass Concrete. Case Stud. Constr. Mater..

[13] Rashad A.M. (2015). A Brief on High-Volume Class F Fly Ash as Cement Replacement—A Guide for Civil Engineer. Int. J. Sustain. Built Environ..

[14] Agwa I.S., Mostafa S.A., Abd-Elrahman M.H., Amin M. (2025). Effect of Recycled Aggregate Treatment Using Fly Ash, Palm Leaf Ash, and Silica Fume Slurries on the Mechanical and Transport Properties of High-Strength Concrete. J. Build. Eng..

[15] Shi C., Jiménez A.F., Palomo A. (2011). New Cements for the 21st Century: The Pursuit of an Alternative to Portland Cement. Cem. Concr. Res..

[16] Lee K.M., Lee H.K., Lee S.H., Kim G.Y. (2006). Autogenous Shrinkage of Concrete Containing Granulated Blast-Furnace Slag. Cem. Concr. Res..

[17] Zhou H., Wu X., Han C., Wei Y. (2025). Research on Durability and Multi-Index Evaluation of Carbon Emissions for High-Strength Concrete with Fly Ash Manufactured Sand. Case Stud. Constr. Mater..

[18] Song B., Hu X., Liu S., Shi C. (2022). Chloride binding of early CO_2_-cured Portland cement-fly ash-GGBS ternary pastes. Cem. Concr. Compos..

[19] Gong K., White C.E. (2016). Impact of Chemical Variability of Ground Granulated Blast-Furnace Slag on the Phase Formation in Alkali-Activated Slag Pastes. Cem. Concr. Res..

[20] Buyondo K.A., Olupot P.W., Kirabira J.B., Yusuf A.A. (2020). Optimization of Production Parameters for Rice Husk Ash-Based Geopolymer Cement Using Response Surface Methodology. Case Stud. Constr. Mater..

[21] Hamada H.M., Al-Attar A.A., Tayeh B., Bin Mat Yahaya F., Abu Aisheh Y.I. (2023). Optimising Concrete Containing Palm Oil Clinker and Palm Oil Fuel Ash Using Response Surface Method. Ain Shams Eng. J..

[22] Shi X., Zhang C., Wang X., Zhang T., Wang Q. (2022). Response Surface Methodology for Multi-Objective Optimization of Fly Ash-GGBS Based Geopolymer Mortar. Constr. Build. Mater..

[23] (2023). Common Portland Cement.

[24] (2017). Fly Ash Used for Cement and Concrete.

[25] (2017). Ground Granulated Blast Furnace Slag Used for Cement, Mortar and Concrete.

[26] (2022). Sand for Construction.

[27] (2016). Standard for Test Method of Performance on Ordinary Fresh Concrete.

[28] (2019). Standard for Test Method of Concrete Physical and Mechanical Properties.

[29] (2020). Test Code for Hydraulic Concrete.

[30] Le T.H., Mai D.L., Ta T.H. (2025). Effects of Fly Ash and Ground Bottom Ash from Thermal Power Plants on Workability, Compressive Strength and Durability of High Performance Fine-Grained Concrete. Appl. Eng. Sci..

[31] Masood S., Lu D., Onyelowe K.C., Shah M.M., Almujibah H., Rezzoug A., Ahmed H., Ramzan T., Ben Kahla N., Ghazouani N. (2025). Performance and Sustainability in Hybrid Concrete: A Study of Recycled Aggregates and Activated Fly Ash. Ain Shams Eng. J..

[32] Hefni Y., Zaher Y.A.E., Wahab M.A. (2018). Influence of Activation of Fly Ash on the Mechanical Properties of Concrete. Constr. Build. Mater..

[33] Singh R.P., Vanapalli K.R., Jadda K., Mohanty B. (2024). Durability Assessment of Fly Ash, GGBS, and Silica Fume Based Geopolymer Concrete with Recycled Aggregates against Acid and Sulfate Attack. J. Build. Eng..

[34] Sandra N., Kawaai K., Ujike I., Thamrin R., Tanjung J., Nsama W., Caronge M.A. (2025). Sustainable Concrete Using Copper Slag and Fly Ash: Microstructural Durability and Corrosion Risk in Vertically Cast Elements. Structures.

